# Gut microbiota and metabolomics unveil the mechanisms of *Lomatogonium rotatum* in ameliorating visceral fat and serum lipids in high-fat diet-induced obese mice

**DOI:** 10.3389/fphar.2024.1418063

**Published:** 2024-11-04

**Authors:** Xiaoping Ji, Hongzhen Yu, Lianqian Wang, Xuemei Bao, Tegele Si, Xiaoman Li, Hugejiletu Wang, Almaz Borjigidai, Galih Kusuma Aji, Laxinamujila Bai, Minghai Fu

**Affiliations:** ^1^ Key Laboratory of Tropical Translational Medicine of Ministry of Education, Hainan Provincial Key Laboratory for Research and Development of Tropical Herbs, School of Pharmacy, Hainan Medical University, Haikou, China; ^2^ College of Traditional Chinese Medicine, Liaoning University of Traditional Chinese Medicine, Shenyang, China; ^3^ Key Laboratory of Ethnomedicine of Ministry of Education, Center on Translational Neuroscience, School of Pharmacy, Minzu University of China, Beijing, China; ^4^ NMPA Key Laboratory for Quality Control of Traditional Chinese Medicine (Mongolian Medicine), School of Mongolian Medicine, Inner Mongolia Minzu University, Tongliao, China; ^5^ Research Center for Agroindustry, National Research and Innovation Agency, Jakarta Pusat, Indonesia

**Keywords:** *Lomatogonium rotatum*, visceral fat, serum lipid, gut microbiota, metabolomics

## Abstract

*Lomatogonium rotatum* (LR) is a folk medicinal herb traditionally used as a lipid-lowering and anti-obesity agent; but its pharmacological mechanism is unclear. In this study, we assessed the alterations of LR on gut microbes and serum metabolites in obese mice and their associated mechanisms of modulation on visceral fat and serum lipid by integrating gut microbiota and metabolomics analyses. Mice were fed a high-fat diet (HFD) to generate obesity and were then given LR and Orlistat orally at different doses (0.18, 0.9, 1.8 g/kg for LR and 0.048 g/kg for Orlistat) for a duration of 9 weeks. The impact of LR on weight loss was assessed through the examination of fat deposition, serum lipid indices, liver indices, and HE pathohistology. The effects of LR on gut microbiota and serum metabolites in obese mice were then investigated by 16S rRNA sequencing technology and untargeted metabolomics, and correlation analysis was performed. LR significantly reduced body weight, feed intake, Lee’s index, visceral fat accumulation, serum TG, TC, AST and ALT, and elevated serum HDL levels in obese mice. In addition, 16S rRNA sequencing results indicated that the LR intervention remodeled microbial diversity and composition, increased the relative abundance of gut microbes *Bacteroidetes* and *Porphyromonadaceae* in HFD-induced obese mice, and decreased the *Deferribacteres*, *Firmicutes* and the *Firmicutes/Bacteroidetes* ratio. Correlation analyses showed that LR regulation of L-tyrosine and hesperetin metabolism, as well as alterations in the metabolic pathways of Phenylalanine, tyrosine and tryptophan biosynthesis, were associated with the changes in abundance of *Bacteroidetes*, *Firmicutes, Porphyromonadaceae* and *Deferribacteres*. Our study demonstrated that LR has lipid lowering and visceral fat reduction effects and its function may be closely related to the improvement of the gut microbiota and its associated metabolites.

## 1 Introduction

The complex interplay between visceral fat, blood lipid levels, and the onset of several diseases has been extensively investigated (Sukkriang et al., 2021). Visceral fat, located deep in the abdomen and around important organs, is now recognized as a significant factor in the complex relationship between metabolism and cardiovascular health (Vasamsetti et al., 2023). Concurrently, deviations in blood lipid profiles, marked by increased levels of cholesterol and triglycerides, have been recognized as significant factors in the initiation and advancement of conditions such as cardiovascular illnesses, diabetes, and metabolic syndrome (Guan et al., 2020). The association between visceral fat accumulation and adverse alterations in blood lipid profiles is multifaceted. Visceral fat is metabolically active and secretes bioactive molecules known as adipokines, which influence lipid metabolism and inflammation (Gugliucci, 2022). The intricate interplay between visceral fat and blood lipids creates a milieu conducive to the development of atherosclerosis, insulin resistance, and chronic inflammation, all precursors to a spectrum of diseases that pose a significant public health challenge (Lechner et al., 2020).

Given the escalating prevalence of these conditions globally, there is an increasing interest in exploring alternative therapeutic approaches. Traditional herbal medicine, with its rich history and holistic philosophy, presents a promising outlook for the treatment and management of visceral fat accumulation and dyslipidemia (Li L et al., 2020; Fu et al., 2022). The potential benefits of herbal remedies lie not only in their historical use but also in their diverse bioactive compounds that exhibit anti-inflammatory, antioxidant, and lipid-lowering properties (Fan and Pedersen, 2021). *Lomatogonium rotatum* (L.) Fries ex Nym (LR), known as Habirigen Digda in Mongolian, was incorporated into the Drug Standard of the Ministry of Health of the People’s Republic of China: Mongolian Drugs Sub-register in 1998 (Dai et al., 2023). It possesses the therapeutic properties of calming “Xila,” clearing heat, strengthening the stomach, and promoting wound healing (Ji et al., 2023). Our recent study revealed that LR extract mitigates diabetes mellitus induced by a high-fat, high-sugar diet and streptozotocin in rats (Dai et al., 2023). In addition, the main constituents of LR, including swertiamarine, sweroside, hesperetin, and several flavonoids, exhibit varying degrees of hepatoprotective and choleretic activities and treat obesity-related disorders (Xu et al., 2021; Kumari et al., 2023; Jayaraman et al., 2018). These include alleviating hypercholesterolemia and hypertriglyceridemia induced by high-fat diet and improving lipid and leptin metabolism in insulin-resistant rats (Baiyisaiti et al., 2019), which laid a preliminary foundation for our research. However, the potential pharmacological mechanism of LR in its anti-adipogenic and lipid-modulating properties remains to be explored.

The intricate relationship between the gut microbiome and human health has gained increasing attention in recent years (Fan et al., 2021). The gut microbiome is a dynamic ecosystem comprised of trillions of bacteria, viruses, fungi, and other microorganisms (Schmidt et al., 2018). Emerging research has uncovered the profound impact of the gut microbiome and its metabolites on metabolic processes, particularly in the context of visceral fat accumulation, serum lipid levels, and obesity (Liu et al., 2021). Dysbiosis may lead to an imbalance in the production of metabolites, impacting host adipose tissue and lipid metabolism (Liu et al., 2017). Studies have suggested that the gut microbiome may impact fat storage and distribution through various mechanisms. This includes the regulation of adipocyte differentiation, lipid metabolism, and the expression of genes involved in fat storage (Cheng et al., 2022). Additionally, the influence of gut microbiota on gut barrier integrity and permeability can contribute to the translocation of microbial products into the bloodstream, potentially triggering inflammatory responses that impact adipose tissue (Kang et al., 2022). The modulation of serum lipid levels by the gut microbiome is another area of active investigation. Research has shown that specific microbial taxa and their metabolites can influence lipid absorption, synthesis, and transport (Fu et al., 2015). For instance, *Bacteroidetes* and *Firmicutes* are involved in bile acid metabolism, which plays a crucial role in lipid digestion and absorption. Dysregulation of these processes can lead to alterations in serum lipid profiles, contributing to dyslipidemia observed in obesity (Magne et al., 2020). Therefore, the gut microbiome and its metabolites play a multifaceted role in modulating visceral fat accumulation, serum lipid levels, and obesity. Understanding the intricate interplay between the gut microbiome and host metabolism holds promise for developing targeted interventions and therapeutic strategies to address lipid disorders and promote overall health. In this study, the effect of LR on microbiome-associated metabolic changes in HFD-induced obese mice were studied by 16S rRNA gene sequencing coupled with LC-MS-based metabolomics technology.

## 2 Materials and methods

### 2.1 Materials and reagents

LR was collected from Xiwuqi, Xilin Gol League, Inner Mongolia, and was authenticated as the dried whole herb of the gentian family LR by Prof. Bagenna of the Mongolian Medical College of Inner Mongolia University for Nationalities. High-fat diet was purchased from Shandong Hengrong Biotechnology Co., Ltd. (China), normal diet was purchased from Liaoning Changsheng Biotechnology Co., Ltd. (China). Serum triglycerides (TG), total cholesterol (TC), high-density lipoprotein (HDL), low-density lipoprotein (LDL), alanine aminotransferase (ALT), and aspartate aminotransferase (AST) kits were purchased from Icubio BioTechnology Ltd., Shenzhen (China). Hematoxylin and eosin (H&E) stains were purchased from Nanjing Jianjian Science and Technology Co. Ltd. (China).

### 2.2 Laboratory animals

A total of sixty male Kunming mice, aged 5 weeks and with a body mass of around 20 ± 2 g, were acquired from Liaoning Changsheng Biotechnology Co. Ltd. (batch No. SCXK(Liao)2015-0001). The animal study protocols underwent assessment and approval by the Ethics Committee of Inner Mongolia Minzu University (Ethics number: NM-LL-2021-06-15-1). The animals were kept in controlled settings with a 12-hour light and dark cycle, at a consistent temperature of 24.0°C ± 2.0°C, and a relative humidity of 50% ± 5%. Prior to conducting the experiments, the mice were acclimated to the setting for a duration of 1 week.

### 2.3 Preparation of LR extract

A total of 5 kg of LR was subjected to the processes of washing, drying, and pulverization. Subsequently, the LR powder was subjected to three consecutive reflux extractions using dichloromethane with each extraction lasting 3 h. The extract underwent a process of combination and concentration by rotary evaporation instrument (RE-2010). Then, 95% ethanol was added to the filter residue and extracted twice (3 h each time). At the end, all extractions were combined and freeze-dried at a temperature of 60 C (FD-1A-50 Freeze Dryer, Beijing BoMedicom Experimental Instrument Co., Ltd.). The LR extract was suspended using carboxymethylcellulose sodium salt (CMC-Na) solvent and administered orally to rats at doses of 0.18 g/kg, 0.9 g/kg, and 1.8 g/kg.

### 2.4 UPLC-MS/MS chemical determination

The chemical profiles of LR extract was analyzed by an ultra-high performance liquid chromatography combining with spectrometry (UPLC-MS/MS) method. A 100 mg of the LR extract was dissolved in 1 mL 80% methanol. The solution was centrifuged and its supernatant was added with 10 μL internal standard, and then filtered through a 0.22 μm filter membrane. Chromatographic separation was performed using Thermo Vanquish UHPLC (Thermo Fisher Scientific). A Zorbax Eclipse C18 column (1.8 μm*2.1*100 mm) was used with a flow rate of 0.3 mL/min at 30°C. The injection volume was 2 μL. The mobile phase consisted of 0.1% formic acid (A) and acetonitrile (B). Gradient elution was applied as follows: 5% B at 0-2 min, 30% B at 2-7 min, 78% B at 7-14 min, 95% B at 14-18 min. Q-Exactive high-resolution mass spectrometer was used for analysis. Electrospray voltage was 3.5 KV; heater temperature and capillary temperature were 325°C and 330°C; sheath gas flow rate, auxiliary gas flow rate and purge gas flow rate were 45 arb, 15 arb and 1 arb respectively; S-Lens RF Level was 55%. Scanning mode was first-level full scan (Full Scan, m/z 100–1500) and data-dependent second-level mass spectrometry scan (dd-MS2, TopN = 10). The collision mode was high energy collision dissociation (HCD). Compound Discoverer 3.2 system was used to perform data acquisition and analysis. The chemical structures were identified using the Thermo mzCloud online database and the Thermo mzValut local database according to the secondary mass spectrometry information.

### 2.5 Experimental design

Sixty SPF grade Kunming mice were acclimatized for 1 week and subsequently randomly assigned into two groups. Ten mice were designated for the normal diet group (ND), while the remaining mice were subjected to a high-fat diet to induce obesity, with *ad libitum* access to both water and food. Following an 8-week period, the obesity model was established, utilizing the criterion defined as an average body weight increase exceeding 20% than the ND mice. Upon successful modeling, the obesity group was randomly subdivided into the high-fat diet group (HFD), LR high, medium, and low dose groups (0.18, 0.9, 1.8 g/kg), and a positive control group (orlistat, 0.048 g/kg), each comprising 10 animals. Throughout a 9-week intervention period, ND and HFD groups received equal volumes of CMC-Na via daily gavage. Weekly observations and recordings of mouse body mass and food intake were conducted. Following the last administration, mice underwent a 12-hour fast without water, followed by weight measurement. Subsequently, 10% chloral hydrate (3 mL/kg) intraperitoneal anesthesia was administered to facilitate body length measurement, determination of the obesity index (Lee’s index), and the collection of serum, mesenteric fat, perirenal fat, epididymal fat, liver tissues, and colonic portion fecal samples. The collected liver and fat tissues were weighed and fixed in paraformaldehyde fixative solution or promptly frozen in liquid nitrogen and stored at −80 C for later analysis.

### 2.6 Biochemical analysis of serum and liver indices

Serum levels of TG, TC, LDL, HDL, ALT, and AST (Icubio BioTechnology Ltd., Shenzhen) were measured by using Varioskan™ LUX Multifunctional Microplate Reader (Thermo Fisher Scientific-CN).

### 2.7 HE staining to observe the pathological changes of liver tissue and adipose tissue

The liver, mesenteric fat, perirenal fat, and epididymal fat tissues were fixed in a 10% paraformaldehyde solution and then dehydrated using a gradient of ethanol concentrations (80%, 90%, 95%, 95%, 95%, and 100%). After being cleared with xylene, the tissues were then fixed in paraffin. Following that, they were sliced into sections that were 3 μm thick and mounted onto slides. The slices were subsequently exposed to a one-hour baking procedure at a temperature of 62 C. The paraffin sections underwent a series of rinses in xylene, anhydrous ethanol, and ethanol solutions of varying concentrations (95%, 90%, 80%), and then were rinsed with water. The sections were submerged in hematoxylin for a duration of 1-3 min and thereafter subjected to eosin staining for a period of 30 s. The samples were securely closed using a coverslip. The liver and adipose tissues were observed for their cellular structure and pathological deterioration using microscopic examination and photography. The nuclei were dyed with a blue stain, while different intensities of red indicated the cytoplasm and other tissue components.

### 2.8 16S rRNA gene sequencing and microbiota analysis

Each fecal sample from the cecum was collected and immediately frozen at −80 C for preservation. Genomic DNA extraction was carried out using the CTAB technique. Amplification of the 16S rRNA genes (16S V3-V4) was performed using custom-designed primers (341F: 5′-CCTAYGGGRBGCASCAG-3′ and 806R: 5′-GGACTACNNGGGTATCTAAT-3′). The PCR amplification products were mixed, purified, and utilized for constructing sequencing libraries with the TruSeq^®^ DNA PCR-Free Sample Preparation Kit. Sequencing was conducted on the NovaSeq6000 platform (Metware Technology Co., Ltd., Wuhan, China) after quality assessment by Qubit 2.0 and q-PCR. Offline data were sorted for each sample based on barcode and PCR amplified primer sequences, which were subsequently removed. FLASH (V1.2.7) was employed to splice reads and obtain raw tags. QIIME (v1.9.1) facilitated rigorous filtration and processing, generating clean reads. The removal of chimeric sequences resulted in the acquisition of final effective tags. Uparse (Uparse v7.0.1001) clustered effective tags, grouping sequences with a similarity exceeding 97% into operational taxonomic units (OTUs). Species annotations for OTU sequences were conducted using the Silva Database SSUrRNA (http://www.arb-silva.de/) and the Mothur algorithm. Alpha and beta diversity analyses were performed using QIIME (v1.9.1) and visualized with R software (v2.15.3). Phylum and genus analyses were conducted through metastats analysis using the R program (v2.15.3).

### 2.9 Serum metabolomics analysis

The serum samples, which were held at a temperature of −80°C in the refrigerator, were defrosted on ice and vigorously mixed for a duration of 10 s. A 50 μL portion of the sample and 300 μL of extraction solution (a mixture of acetonitrile and methanol in a ratio of 1:4, volume to volume), which includes internal standards, were mixed together in a 2 mL microcentrifuge tube. The mixture was subjected to vortexing for a duration of 3 min, followed by centrifugation at a speed of 12,000 revolutions per minute for a duration of 10 min at a temperature of 4°C. Afterwards, 200 μL of the liquid remaining after centrifugation was gathered and stored at a temperature of −20°C for a duration of 30 min. This was then followed by another round of centrifugation at a speed of 12,000 revolutions per minute for a duration of 3 min at a temperature of 4°C. A 180 μL portion of the supernatant was transferred for LC-MS analysis at Metware Technology Co., Ltd. in Wuhan, China. The LC-MS system was used to process all samples in accordance with the machine’s instructions. The UPLC analysis was performed using a Waters ACQUITY UPLC HSS T3 C18 column (1.8 µm, 2.1 mm*100 mm) at a temperature of 40°C. The flow rate was set at 0.4 mL/min and the injection volume was 2 μL. The solvent system consisted of water (0.1% formic acid) and acetonitrile (0.1% formic acid) with a gradient program. The gradient program started with a ratio of 95:5 V/V at 0 min, followed by a change to 10:90 V/V at 11.0 min, maintaining this ratio until 12.0 min. Finally, the ratio was switched back to 95:5 V/V at 12.1 min and maintained until 14.0 min.

Unsupervised principal component analysis (PCA) was performed using the prcomp function in R (www.r-project.org). Prior to unsupervised PCA, the data underwent unit variance scaling. The identification of metabolites that were significantly regulated across groups was based on the following criteria: a Variable Importance in Projection (VIP) score of ≥1, a fold change (FC) of ≥1.2 or ≤0.833, and a *p*-value of <0.05 (Cao et al., 2022). The VIP (Variable Importance in Projection) values were derived using the findings of OPLS-DA (Orthogonal Partial Least Squares Discriminant Analysis). These results, which encompassed score plots and permutation plots, were obtained using the R package MetaboAnalystR. Prior to OPLS-DA, the data underwent log transformation (base 2) and mean centering. In order to mitigate the risk of overfitting, a permutation test consisting of 200 permutations was conducted. The identified metabolites were annotated utilizing the KEGG Compound database, accessible at http://www.kegg.jp/kegg/compound/. The metabolites with annotations were further analyzed using MetaboAnalyst 5.0 (http://www.metaboanalyst.ca/) (Pang et al., 2021) to identify probable metabolic pathways.

### 2.10 Statistical analysis

The data were presented as the mean ± SEM. The data was analyzed using one-way analysis of variance (ANOVA) to compare among groups. The two groups were compared using a *t*-test. The Graphpad Prism 8.0 and MetwareCloud Platform were utilized for generating graphics. A *p*-value less than 0.05 was considered to be statistically significant.

## 3 Results

### 3.1 HPLC determination of main compounds in LR extract

Based peak intensity (BPI) chromatograms of LR extract was acquired by LC-MS/MS. After identification, a total of 48 and 81 compounds were identified in the negative and positive ion modes, respectively. Among them, 9 compounds with relative percentages higher than 2% were listed in Table 1. Swertiamarin has the highest content, accounting for 30.39%. The total inclusion rate of the 9 identified compounds reached 86.26% in the LR extract.

**TABLE 1:** Main compounds of LR extract by LC-MS/MS determination.

No	Ion mode	Compound name	Formula	PubChem ID	RT/min	Annotation MW	Calc. MW	Relative percentage (%)
1	negative	Swertiamarin	C_16_H_22_O_10_	442,435	5.414	374.121	374.121	30.39%
2	negative	α,α-Trehalose	C_12_ H_22_ O_11_	7,427	0.793	342.116	342.116	16.64%
3	negative	2- {[4-(4-Fluorophenyl)-6-(trifluoromethyl)-2-pyrimidinyl]sulfanyl}-N-(2-phenylethyl)acetamide	C_21_ H_17_ F_4_ N_3_ OS	3144676	7.401	435.103	435.102	10.50%
4	negative	Bellidifolin	C_14_ H_10_ O_6_	5281623	11.583	274.048	274.047	9.95%
5	negative	(1S,4aS,7S,7aS)-7-Hydroxy-1-{[6-O-(4-hydroxybenzoyl)-β-D-glucopyranosyl]oxy}-7-methyl-1,4a,5,6,7,7a-hexahydrocyclopenta [c]pyran-4-carboxylic acid	C_23_ H_28_ O_12_	NA	7.102	496.159	496.158	5.56%
6	negative	Sweroside	C_16_ H_22_ O_9_	161,036	5.842	358.126	358.126	4.01%
7	negative	Alpha-tyvelopyranosyl-(1->3)-alpha-D-mannopyranose	C_12_ H_22_ O_9_	45266831	2.085	310.126	310.126	3.49%
8	negative	Mussaenosidic acid	C_16_ H_24_ O_10_	1633105	4.855	376.137	376.136	3.42%
9	negative	Amarogentin	C_29_ H_30_ O_13_	115,149	7.900	586.170	586.168	2.30%

### 3.2 Effects of LR on body weight, Lee’s index and feed intake in HFD-fed mice

**FIGURE 1:**
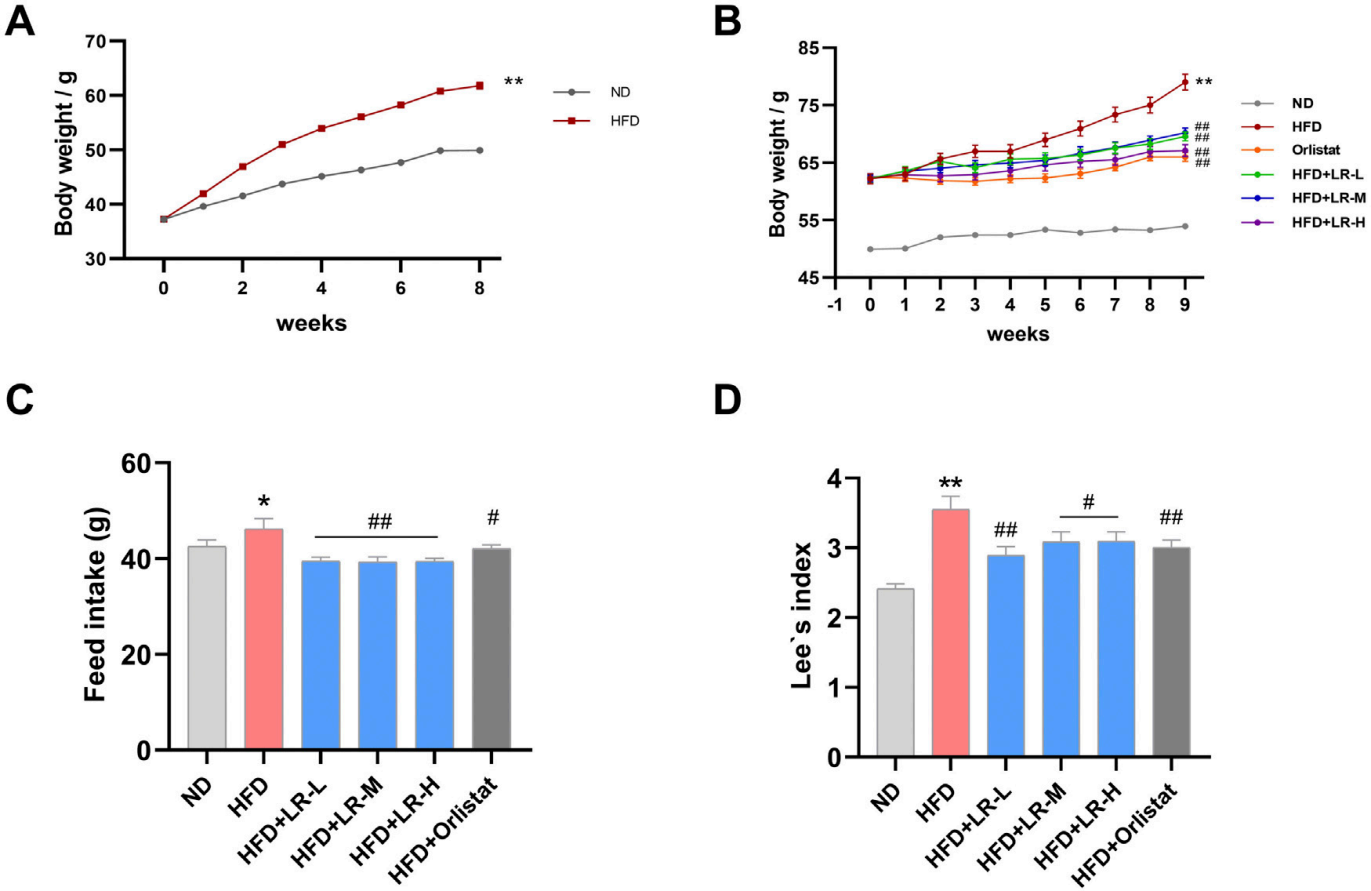
Obese mice modeling **(A)** and the evaluation of LR effects on body weight **(B)**, feed intake **(C)** and Lee’s index **(D)**. Data were presented as mean ± SEM (n = 10). ^*^ *p* < 0.05, ^**^ *p* < 0.01 vs. ND, ^#^ *p* < 0.05, ^##^ *p* < 0.01 vs. HFD.

As depicted in Figure 1A, there was a significant increase in body weight observed in mice subjected to a high-fat diet compared to those on a normal diet after an 8-week modeling period. Subsequently, either LR or orlistat was orally administered for an additional 9 weeks to assess their impact on anti-obesity indices. The results revealed a significant elevation (*p* < 0.05) in body weight, feed intake, and Lee’s index in the HFD group compared to the ND group (Figures 1B–D, respectively). However, administration of LR and orlistat markedly reduced (*p* < 0.05) these values.

### 3.3 Effects of LR on serum lipids, liver function indices and liver histology in HFD-fed mice

**FIGURE 2:**
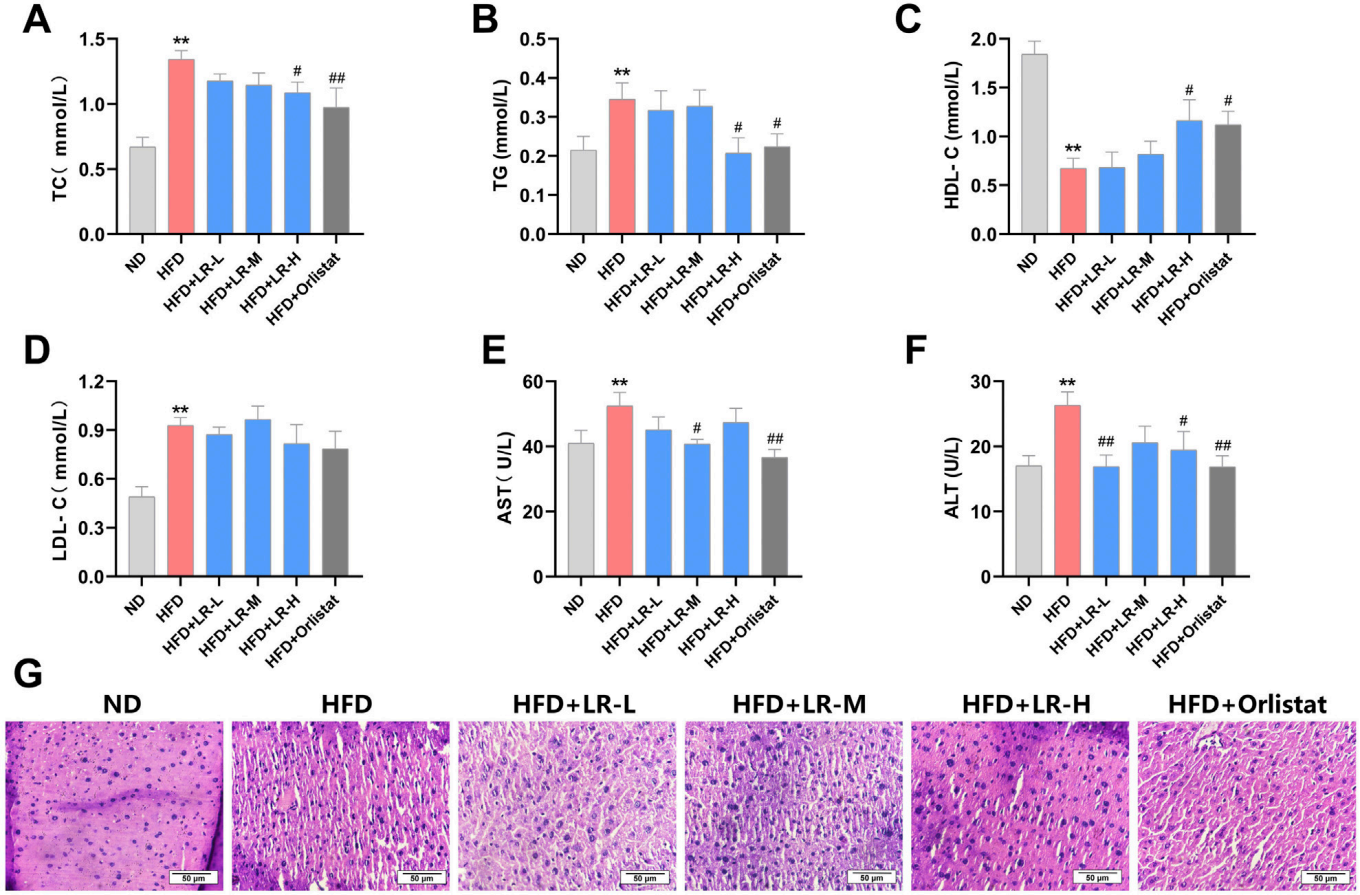
Effects of LR on lipid serum lipids **(A–D)**, liver function indices **(E, F)** and liver histology **(G)** in obese mice. Data were presented as mean ± SEM (n = 10). ^*^ *p* < 0.05, ^**^ *p* < 0.01 vs. ND, ^#^ *p* < 0.05, ^##^ *p* < 0.01 vs. HFD.

As illustrated in Figures 2A–D, the levels of TC, TG and LDL-C exhibited a significant increase, while HDL-C showed a significant decrease in the HFD group compared to the ND group (*p* < 0.05). Administration of LR-H and orlistat significantly (*p* < 0.05) mitigated serum TC and TG levels, and concurrently increased HDL levels in mice compared to the HFD group. No changes were observed in LDL levels after LR treatment. In addition, AST and ALT levels were significantly elevated in the HFD group compared to the ND group. Treatment with LR-M and orlistat significantly (*p* < 0.05) decreased AST levels, and LR-L and LR-H decreased ALT levels in mice (Figures 2E, F).

Morphological analysis of liver tissue revealed that the structure and size of hepatocytes in the control group remained unchanged, while the cellular morphology and boundaries of hepatocytes in the HFD group were unclear and exhibited severe damage. Treatment with LR extract and orlistat resulted in clear hepatocyte structure and significantly improved cellular morphology, with the LR-H group showing particularly pronounced effects (Figure 2G).

### 3.4 Effect of LR on visceral adipose tissue in HFD-fed mice

**FIGURE 3:**
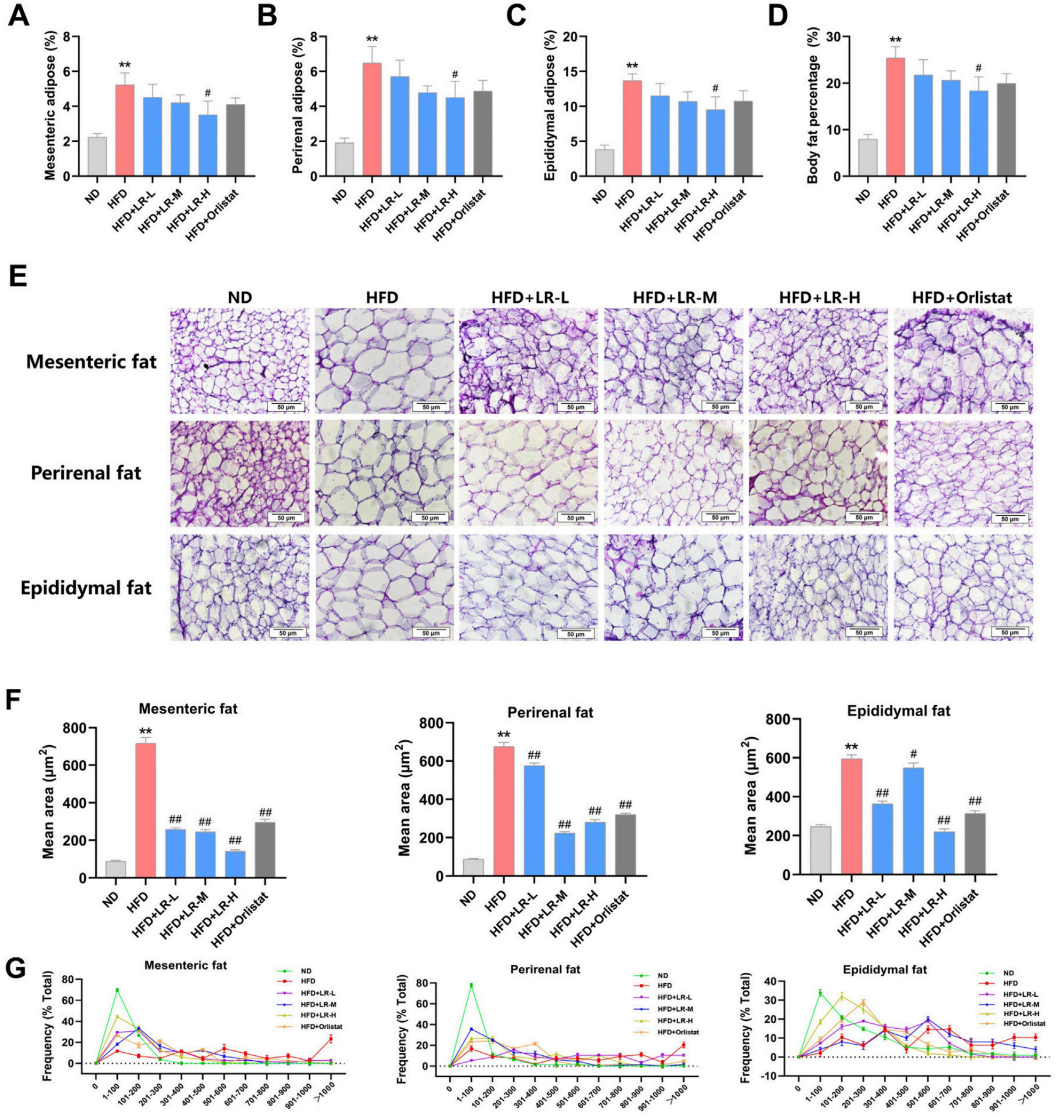
Effect of LR on visceral adipose tissue in obese mice. **(A)** Mesenteric fat; **(B)** Perirenal fat; **(C)** Epididymal fat; **(D)** Body fat percentage; **(E)** H&E staining sections, **(F)** Average size and **(G)** Size distribution of mesenteric fat, perirenal fat and epididymal fat tissues. The fat tissue percentage was calculated as fat weight/bodyweight x 100%. The size distribution and average size of each fat tissue were analyzed using Image J. Data were presented as mean ± SEM (n = 10). *p < 0.05, **p < 0.01 vs. ND, #*p* < 0.05, ##*p* < 0.01 vs. HFD.

After examining the weight and structure of the visceral adipose tissue, it was evident that the mesenteric fat, perirenal fat, epididymal fat and size of adipocytes showed a substantial increase in weight in the HFD group compared to the ND group (*p* < 0.01). The adipocytes exhibited hypertrophy and were replete with lipid droplets, concomitant with a marked decrease in the adipocyte count within the same microscopic field of view. Administration of LR through gavage resulted in a notable reduction in the mass of mesenteric fat, perirenal fat, epididymal fat and size of adipocytes (*p* < 0.05). Furthermore, there was a notable decrease in cell width, and a substantial rise in the number of adipocytes observed within the same microscopic field of view (Figures 3A–G). The impact was most noticeable in the LR-H group. These findings are consistent with the results obtained from the examination of serum lipid indices.

### 3.5 Effect of LR on the structure of intestinal flora in HFD-fed mice

**FIGURE 4:**
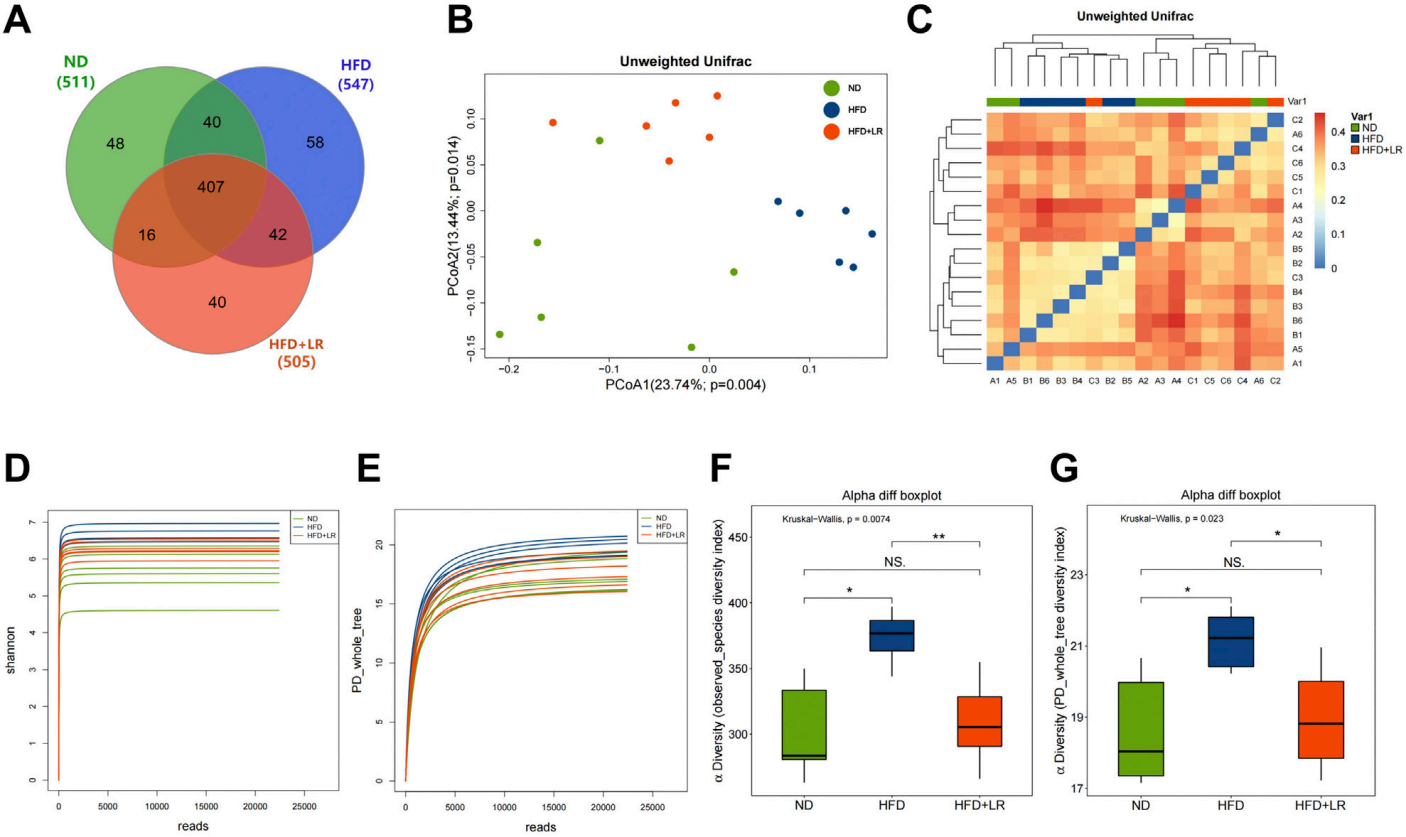
The results of microbial community diversity analysis of gut microbiota in three groups. **(A)** Venn diagram of the shared functional groups; **(B)** PCoA diagram of mouse intestinal microflora; **(C)** OTU-based hierarchical cluster analysis with unweighted group averaging method; **(D)** Rarefaction curves showing the average Shannon number for three groups. **(E)** Rarefaction curves showing the average PD whole tree number for three groups; **(F)** observed species index diversity in three experimental groups; **(G)** PD whole tree diversity in three experimental groups. Data were presented as mean ± SEM (n = 6). ^*^ *p* < 0.05, ^**^ *p* < 0.01 vs. ND, ^#^ *p* < 0.05, ^##^ *p* < 0.01 vs. HFD.

Based on the phenotypic outcomes, the LR-H group (hereinafter referred to as the LR group) exhibited notable improvements in the reduction of obese mice, prompting the selection of the high-dose group for further investigation. Results from the Venn diagram revealed a total of 407 OTUs common to all three groups (62.5%), 447 OTUs shared between the ND and HFD groups, and 449 OTUs shared between the HFD and LR groups (Figure 4A). Diversity analysis demonstrated significant differences and clear separation among samples from the ND, HFD, and LR groups (Figure 4B). As expected, substantial segregation was evident in the evolutionary tree results for samples from the ND, HFD, and LR groups, consistent with the findings of PCoA (Figure 4C). α Diversity analysis indicated that the sequencing depth was sufficient to detect all species in the samples. The observed species and PD whole tree diversity indices were significantly higher (*p* < 0.05) in the HFD group, whereas they were significantly lower (*p* < 0.05) in the LR group (Figures 4D–G).

### 3.6 Effects of LR on structure compositions of gut microbiota and KEGG pathways

**FIGURE 5:**
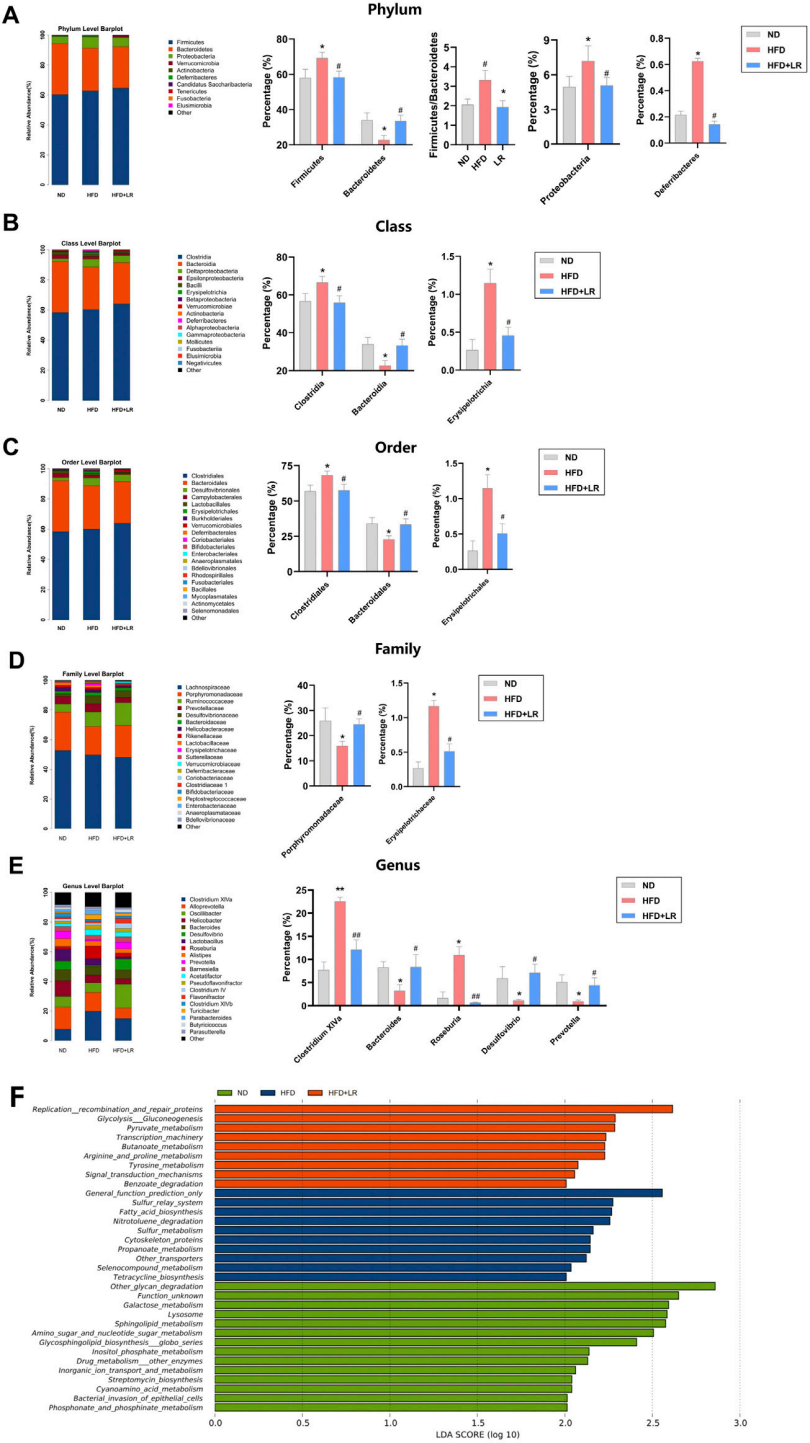
Effect of LR on intestinal flora and its KEGG differential pathways. Relative abundance of gut microbiota at phylum level **(A)**, class level **(B)**, order level **(C)**, family level **(D)** and genus level **(E)**. KEGG differential pathway diagram of ND, HFD and LR groups **(F)**. ND, normal diet group; HFD, high-fat diet group; LR, *Lomatogonium rotatum-*treated group. Data were presented as mean ± SEM (n = 6). ^*^ *p* < 0.05, ^**^ *p* < 0.01 vs. ND, ^#^ *p* < 0.05, ^##^ *p* < 0.01 vs. HFD.

Species annotation results were utilized to generate corresponding bar charts visualizing species profiling at various taxonomic levels for each group, including phylum, class, order, family, and genus. At the phylum level, the mouse gut microbiota exhibited a predominant composition of *Firmicutes* and *Bacteroidetes*. In the HFD group, there was a relative increase in the abundance of *Firmicutes*, *Proteobacteria*, *Firmicutes/Bacteroidetes*, and *Deferribacteres*, coupled with a relative decrease in the abundance of *Bacteroidetes* compared to the ND group. LR administration led to a decrease in *Firmicutes*, *Proteobacteria*, *Firmicutes/Bacteroidetes*, and *Deferribacteres* abundance, and an increase in *Bacteroidetes* abundance (Figure 5A). At the class level, there was a significantly higher abundance of *Clostridia* and *Erysipelotrichia*, and a significantly lower abundance of *Bacteroidia* in the HFD group. These trends were reversed following LR intervention (Figure 5B). At the order level, *Clostridiales* and *Erysipelotrichales* exhibited significantly higher abundance, while *Bacteroidales* exhibited significantly lower abundance in the HFD group. LR intervention led to increased *Bacteroidales* abundance and decreased *Clostridiales* and *Erysipelotrichales* abundance (Figure 5C). At the family level, *Erysipelotrichaceae* abundance was significantly elevated, and *Porphyromonadaceae* abundance was significantly decreased in the HFD group. LR administration resulted in a convergence of *Erysipelotrichaceae* and *Porphyromonadaceae* abundance to levels observed in the ND group (Figure 5D). At the genus level, there was a significant increase in *Clostridium XlVa* and *Roseburia* abundance, and a significant decrease in *Bacteroides*, *Desulfovibrio*, and *Prevotella* abundance in the HFD group. LR intervention decreased *Clostridium XlVa* and *Roseburia* abundance, and increased *Bacteroides*, *Desulfovibrio*, and *Prevotella* abundance (Figure 5E).

LDA Effect Size analysis revealed nine major metabolic pathways in the LR group that produced significantly different effects in the flora between groups, including Replication recombination and repair proteins, Glycolysis Gluconeogenesis, Pyruvate metabolism, Transcription machinery, Butanoate metabolism, Arginine and proline metabolism, Tyrosine metabolism, Signal transduction mechanisms, and Benzoate degradation, among others (Figure 5F).

### 3.7 Effect of LR on serum differential metabolites in HFD-fed mice

**FIGURE 6:**
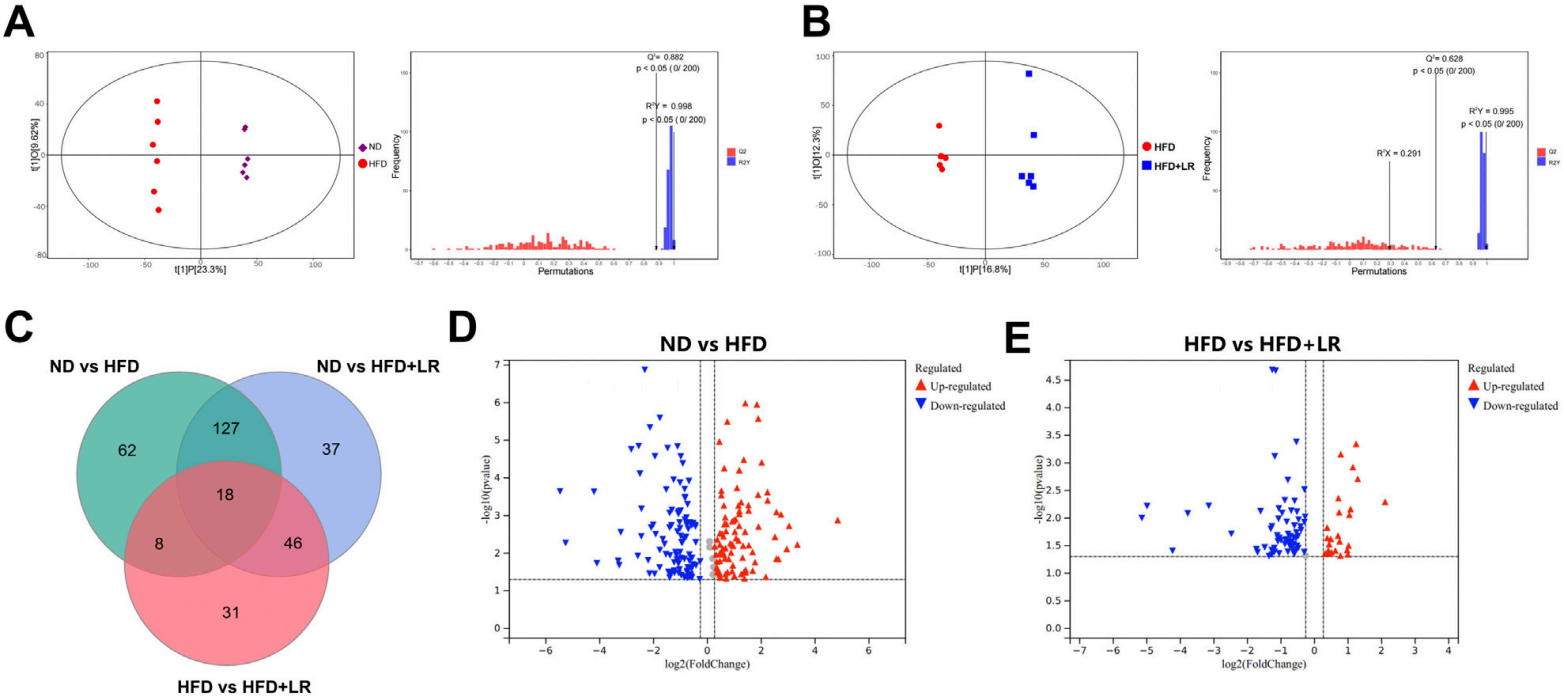
LR altered the associated metabolic profiles in HFD-fed mice. **(A)** OPLS-DA score scatter plot and permutation plot for ND vs. HFD groups. **(B)** OPLS-DA score scatter plot and permutation plot for HFD vs. LR groups. **(C)** Venn diagram of differential metabolites. **(D)** A volcano plot of ND vs. HFD groups. **(E)** A volcano plot of HFD vs. LR groups.

Metabolomics analysis was conducted on serum samples from the ND, HFD, and LR groups. The OPLS-DA results demonstrated a significant separation between the ND and HFD groups (Q2 = 0.882, R2Y = 0.998), as well as between the HFD and LR groups (Q2 = 0.628, R2Y = 0.995) with a *p*-value <0.05. The regression curves validated the credibility of the models (Figures 6A, B). Based on the OPLS-DA outcomes, VIP values were calculated to assess the contribution of metabolites. Metabolites with VIP>1 and *p* < 0.05 were considered statistically significant. Analysis of differential metabolites identified a total of 329 metabolites in the three sets of samples (Figure 6C). Volcano plot analysis of differential metabolites revealed 215 metabolites with significant differences between the ND and HFD groups, with 103 upregulated and 112 downregulated in the HFD group (Figure 6D). Additionally, 103 metabolites exhibited significant differences between the HFD and LR groups, with 24 upregulated and 79 downregulated in the LR group (Figure 6E).

**FIGURE 7:**
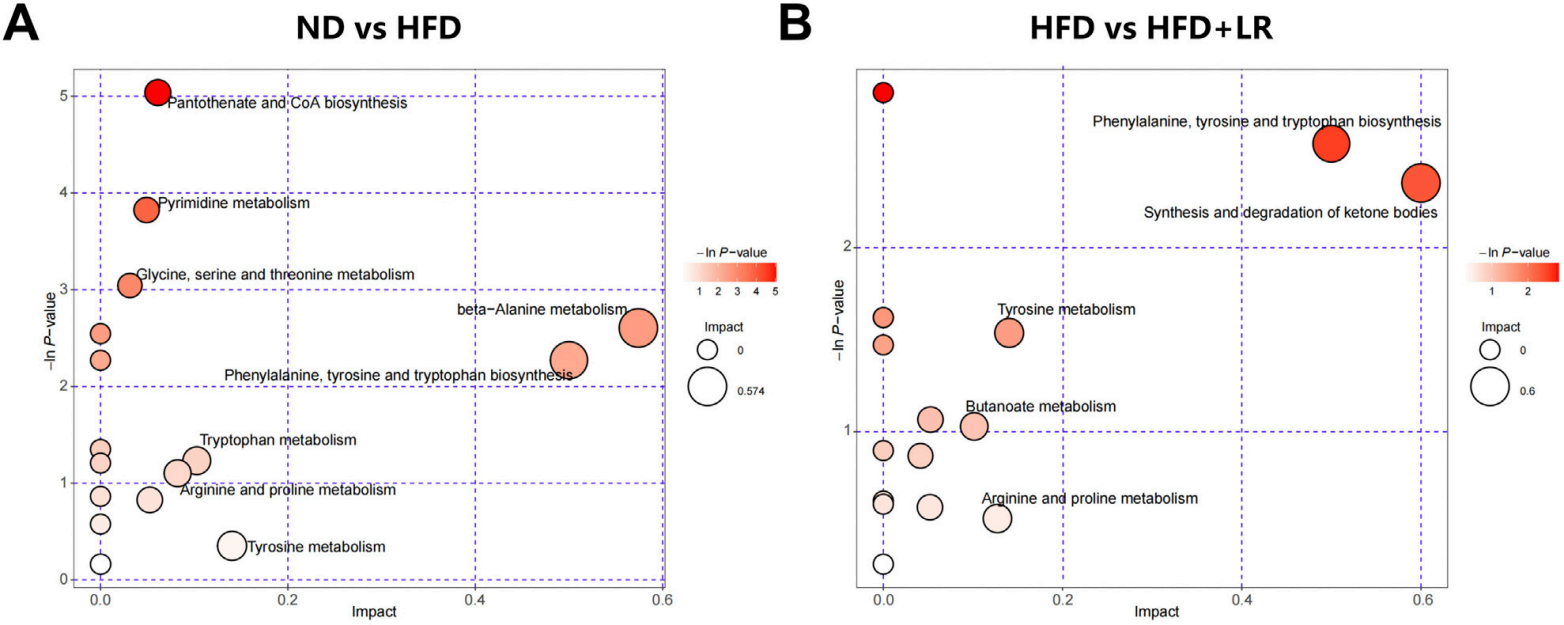
Metabolic pathway analysis of differential metabolites in HFD-fed mice. **(A)** ND vs HFD group; **(B)** HFD vs LR group.

Table 2 presents representative differential metabolites following treatment with LR extracts. Compared to the ND group, the serum of mice in the HFD group exhibited significant increases in 2-Hydroxyethanesulfonate, L-tyrosine, pyro-L-glutaminyl-L-glutamine, lysoPC (16:1 (9Z)/0:0), PSF-A, 6′-Apiosyllotaustralin, kynurenic acid, deoxyuridine, thymine, hesperetin, picraquassioside A, and 4-Hydroxy-5-phenyltetrahydro-1,3-oxazin-2-one, while the levels of proline betaine, sphingosine, and (R)-pelletierine were significantly decreased. LR treatment normalized the levels of these metabolites (*p* < 0.05). The metabolite types encompassed organic acids and derivatives, lipids and lipid-like molecules, carbohydrates, and organoheterocyclic compounds. Metabolic pathway analyses revealed that after LR intervention, 15 metabolic pathways were significantly altered, including phenylalanine, tyrosine, and tryptophan biosynthesis, synthesis and degradation of ketone bodies, tyrosine metabolism, butanoate metabolism, arginine and proline metabolism, ubiquinone and other terpenoid-quinone biosynthesis, phenylalanine metabolism, riboflavin metabolism, nicotinate and nicotinamide metabolism, sphingolipid metabolism, glutathione metabolism, porphyrin and chlorophyll metabolism, valine, leucine, and isoleucine degradation, fatty acid metabolism, and tryptophan metabolism (Figure 7).

**TABLE 2:** Differential metabolites were determined by cross-comparison between different groups.

NO.	Metabolites	Super.class	ND vs HFD				HFD vs HFD + LR			
			VIP	FC	Trend	*P*-value	VIP	FC	Trend	*P*-value
1	2-Hydroxyethanesulfonate	Organic acids and derivatives	1.81	3.20	↓	0.009	2.06	0.58	↑	0.002
2	L-Tyrosine		1.59	1.06	↓	0.005	2.48	0.00	↑	0.002
3	Pyro-L-glutaminyl-L-glutamine		1.63	1.62	↓	0.003	1.63	0.70	↑	0.032
4	LysoPC(16:1 (9Z)/0:0)	Lipids and lipid-like molecules	1.77	1.80	↓	0.005	1.98	0.81	↑	0.003
5	PSF-A		1.59	1.06	↓	0.005	2.48	0.00	↑	0.003
6	6′-Apiosyllotaustralin	Carbohydrates	1.59	1.06	↓	0.005	2.07	0.00	↑	0.026
7	Kynurenic acid	Organoheterocyclic compounds	1.67	3.21	↓	0.029	1.32	0.41	↑	0.016
8	Deoxyuridine		1.70	1.92	↓	0.021	1.58	0.66	↑	0.044
9	Thymine	Nitrogenous bases	1.54	1.38	↓	0.006	1.84	0.69	↑	0.008
10	Hesperetin	Phenylpropanoids and polyketides	1.88	3.58	↓	0.000	2.42	0.01	↑	0.016
11	Picraquassioside A	Organic oxygen compounds	1.25	1.59	↓	0.036	1.43	0.69	↑	0.037
12	4-Hydroxy-5-phenyltetrahydro-1,3-oxazin-2-one	Benzenoids	1.59	1.06	↓	0.005	2.09	0.00	↑	0.017
13	Proline betaine	Amino acids	1.64	0.40	↑	0.001	1.40	1.54	↓	0.039
14	Sphingosine	Organic nitrogen compounds	1.31	0.51	↑	0.039	1.48	1.99	↓	0.045
15	(R)-Pelletierine	Organoheterocyclic compounds	1.34	0.75	↑	0.021	1.55	1.24	↓	0.044

### 3.8 Correlation analysis of the gut microbiota and differential metabolites

**FIGURE 8:**
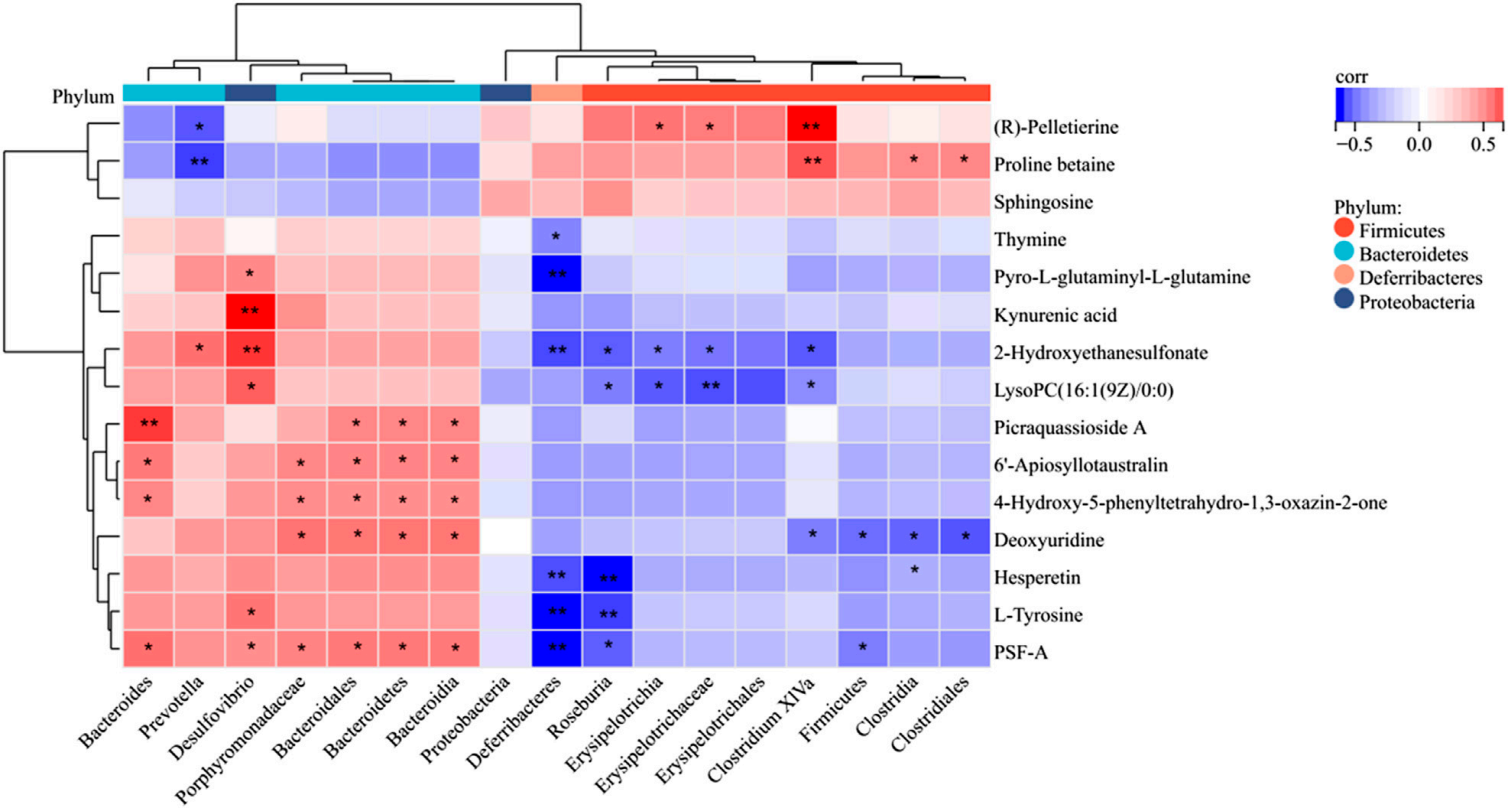
Spearman correlation heatmap of differentially expressed microflora and serum metabolites. The red color represents a positive correlation, while the blue color represents a negative correlation. ^*^ *p* < 0.05; ^**^ *p* < 0.01.

Following the identification of gut differential flora and serum differential metabolite variations in LR-intervened HFD-induced obese mice, a correlation analysis was performed using the Spearman correlation coefficient method (Figure 8). This analysis revealed significant correlations between specific gut flora and metabolites, suggesting potential interactions between microbial composition and metabolic regulation under LR intervention. For instance, the LR treatment led to a decrease in the abundance of *Firmicutes* and *Deferribacteres*. Changes in *Firmicutes* showed a negative correlation with deoxyuridine and PSF-A. Similarly, *Deferribacteres* exhibited negative correlations with a broader range of metabolites, including thymine, pyro-L-glutaminyl-L-glutamine, 2-Hydroxyethanesulfonate, hesperetin, L-tyrosine, and PSF-A. These findings indicate that reductions in these bacterial populations may be linked to the altered metabolism of these specific compounds. In contrast, LR intervention led to an increased abundance of *Bacteroidetes* and *Porphyromonadaceae*. Changes in *Bacteroidetes* were positively correlated with metabolites such as 6′-Apiosyllotaustralin, 4-Hydroxy-5-phenyltetrahydro-1,3-oxazin-2-one, Picraquassioside A, and PSF-A. Similarly, *Porphyromonadaceae* exhibited positive correlations with 6′-Apiosyllotaustralin, 4-Hydroxy-5-phenyltetrahydro-1,3-oxazin-2-one, deoxyuridine, and PSF-A. These findings highlight the complex interactions between the gut microbiome and host metabolism under the influence of LR, pointing to specific microbial shifts that correlate with metabolic changes in HFD-induced obesity.

## 4 Discussion

LR emerges as a multifaceted botanical agent with a substantial impact on both lipid metabolism and the storage of visceral fat, as evidenced by our comprehensive investigation utilizing a HFD-induced obese mice model. Our study demonstrates a pronounced lipid-lowering effect of LR, characterized by significant reductions in serum triglycerides (TG), total cholesterol (TC), as well as aspartate aminotransferase (AST) and alanine aminotransferase (ALT) levels. Concomitantly, LR administration led to an elevation in high-density lipoprotein (HDL) levels, reflecting a favorable shift in lipid profiles associated with improved cardiovascular health. These findings underscore the potential of LR as a therapeutic agent for ameliorating dyslipidemia, a critical component of metabolic disorders. Equally noteworthy is LR’s impact on visceral fat storage. The intervention resulted in a substantial decrease in body weight, feed intake, Lee’s index, and the accumulation of visceral fat in HFD-induced obese mice. The modulation of gut microbiota composition, particularly the increase in the relative abundance of *Bacteroidetes* and *Porphyromonadaceae*, coupled with a decrease in *Deferribacteres* and the *Firmicutes/Bacteroidetes* ratio, aligns with the observed reduction in visceral fat. This suggests a pivotal role of LR in reshaping the gut microbiota landscape, subsequently influencing adipose tissue dynamics and, notably, visceral fat storage.

LR exhibits a diverse chemical composition, and its pharmacological actions are integral to its efficacy in addressing lipid disorders. In this study, we have identified nine main bioactive compounds from LR and swertiamarine accounts for the highest concentration. Swertiamarine is an iridoid glycoside with potent anti-adipogenic and lipid-modulating properties (Dai et al., 2023). It has been suggested to influence lipid metabolism and adipocyte differentiation, potentially leading to a reduction in fat accumulation. Additionally, its antioxidant and anti-inflammatory properties may contribute to its protective effects against obesity-related disorders (Ji et al., 2023; Xu et al., 2021). Similar to swertiamarine, sweroside has been studied for its potential anti-obesity effects (Xu et al., 2021). Furthermore, flavonoids such as coumarin and others, found in various fruits and vegetables, have demonstrated potential anti-obesity effects by influencing adipocyte function and lipid storage, as well as enhancing fat oxidation and thermogenesis (Jayaraman et al., 2018; Baiyisaiti et al., 2019). Consistent with previous reports, the present work further confirmed the pharmacological actions of LR on modulating lipid homeostasis and adipocyte regulation.

In recent years, the idea that gut flora is an important factor in the development of obesity has been widely accepted. When the body is in a normal state of microecological balance between the host and the microbiota, the main reason for this phenomenon is that the intestinal flora maintains a relationship of interdependence and mutual restraints (Fan et al., 2021; Schmidt et al., 2018). When the body is in a healthy state, *Bacteroidetes* and *Firmicute* are dominant in the intestinal tract, accounting for more than 90% of the flora. *Bacteroidetes* and *Firmicutes* have a symbiotic relationship with the body’s anti-obesity mechanisms. They reside in the human body, receiving shelter and nutrients, while also collaborating to enhance the metabolism of polysaccharides. This collaboration leads to increased energy absorption and digestion, ultimately preventing fat accumulation and promoting weight loss. Changes in the proportional abundance of *Bacteroidetes* and *Firmicutes* as marker flora in response to gut microbes in obese populations (Mariat et al., 2009). It has been shown in many studies that obesity dysregulates the gut microbiota, with a decrease in the relative abundance of *Bacteroidetes* and an increase in the relative abundance of *Firmicutes*. And with weight loss *Bacteroidetes* increase while *Firmicutes* decreases (Stojanov et al., 2020). In the present study, we noted a significant increase in the abundance and diversity of the gut microbiota in HFD-induced obese mice, and LR-treated mice showed restored microbiota. The LR intervention also increased the relative abundance of intestinal *Bacteroidetes* in HFD-induced obese mice, decreased the relative abundance of *Firmicutes* and *Firmicutes/Bacteroidetes*, and ameliorated the changes in intestinal flora induced by HFD. Another study indicated a substantial correlation between *Deferribacteres* and *Porphyromonadaceae* and obesity. The abundance of *Deferribacteres* is much higher in obesity models caused by a HFD, but the abundance of *Porphyromonadaceae* is significantly lower (Li Y et al., 2020; Tavella et al., 2021). In this study, LR intervention suppressed enrichment of *Deferribacteres* and increased enrichment of *Porphyromonadaceae* in obese mice; Several metabolic pathways, including Glycolysis Gluconeogenesis, Pyruvate metabolism, Butanoate metabolism, Arginine and proline metabolism, and Tyrosine metabolism, may play key roles in the regulation of intestinal flora by the LR treatment.

In addition, *Bacteroidetes*, *Firmicute*, *Deferribacteres*, and *Porphyromonadaceae* were highly correlated with several serum metabolites. Thymine, pyro-L-glutaminyl-L-glutamine, 2-Hydroxyethanesulfonate, hesperetin, L-Tyrosine, 6′-Apiosyllotaustralin, 4-Hydroxy-5- phenyltetrahydro-1,3, deoxyuridine, Picraquassioside A, deoxyuridine, and PSF-A can potentially differentiate between different types of bacteria such as *Firmicute*, *Bacteroidetes*, *Deferribacteres*, and the intestinal flora metabolites of *Porphyromonadaceae*. Phenylalanine, tyrosine and tryptophan biosynthesis may be one of the major metabolic pathways, and L-Tyrosine is a differential metabolite that regulates this pathway (Wiggins et al., 2015). L-Tyrosine is a natural form of a non-essential amino acid formed from phenylalanine, which helps to replenish norepinephrine and is used in the production of adrenaline and dopamine, two brain chemicals that affect mood and reduce stress. It has been shown that L-Tyrosine is downregulated in obese mice and is negatively correlated with body weight (Hao et al., 2001; Olsson et al., 2021). In the present study, hesperetin was found to be significantly reduced in the HFD group and increased in the LR group, suggesting that LR can replenish the body’s levels of the vitamin hesperetin, thereby reducing lipid accumulation and modulating dyslipidemia (Taheri et al., 2023). Therefore, it is hypothesized that LR may affect the metabolism of L-Tyrosine and hesperetin by regulating the structure of intestinal flora, and participate in the regulation of the metabolic pathways such as phenylalanine, tyrosine and tryptophan biosynthesis, and thus achieve the anti-obesity and lipid-lowering effects. The present study reaffirms that the modulation of obesity by natural medicines is associated with alterations in the composition of the gut microbiota and its metabolites.

The intricate relationship between *Deferribacteres* and the storage of visceral fat has been a subject of considerable interest and investigation in the context of obesity-related pathophysiology (Li et al., 2022; Wu et al., 2020). Our findings reveal a noteworthy association between the abundance of *Deferribacteres*, a taxonomic group within the *Firmicutes* phylum, and the accumulation of visceral fat in HFD-induced obese mice. Furthermore, the observed decrease in the abundance of *Deferribacteres* following LR intervention coincided with a reduction in visceral fat storage. This suggests a potential role of *Deferribacteres* in the regulation of adipose tissue deposition, specifically within the visceral compartment. The *Firmicutes* phylum, to which *Deferribacteres* belongs, has been previously implicated in energy extraction and storage efficiency, making its modulation a pivotal factor in metabolic processes (Murphy et al., 2010). Correlation analyses further underscored the connection between alterations in *Deferribacteres* abundance and specific metabolic pathways associated with weight regulation. These pathways include, but are not limited to, L-Tyrosine and hesperetin metabolism, as well as modifications in the metabolic pathways of Phenylalanine, tyrosine, and tryptophan biosynthesis. Such associations suggest a potential regulatory role of *Deferribacteres* in influencing the metabolic milieu linked to visceral fat storage (Wang et al., 2022; Kindt et al., 2018). While further investigations are warranted to unravel the precise mechanisms underlying the *Deferribacteres*-visceral fat relationship, our study provides compelling evidence of a correlative link, thereby contributing to the evolving understanding of the intricate interplay between gut microbiota components and adipose tissue dynamics in the context of obesity.

**FIGURE 9:**
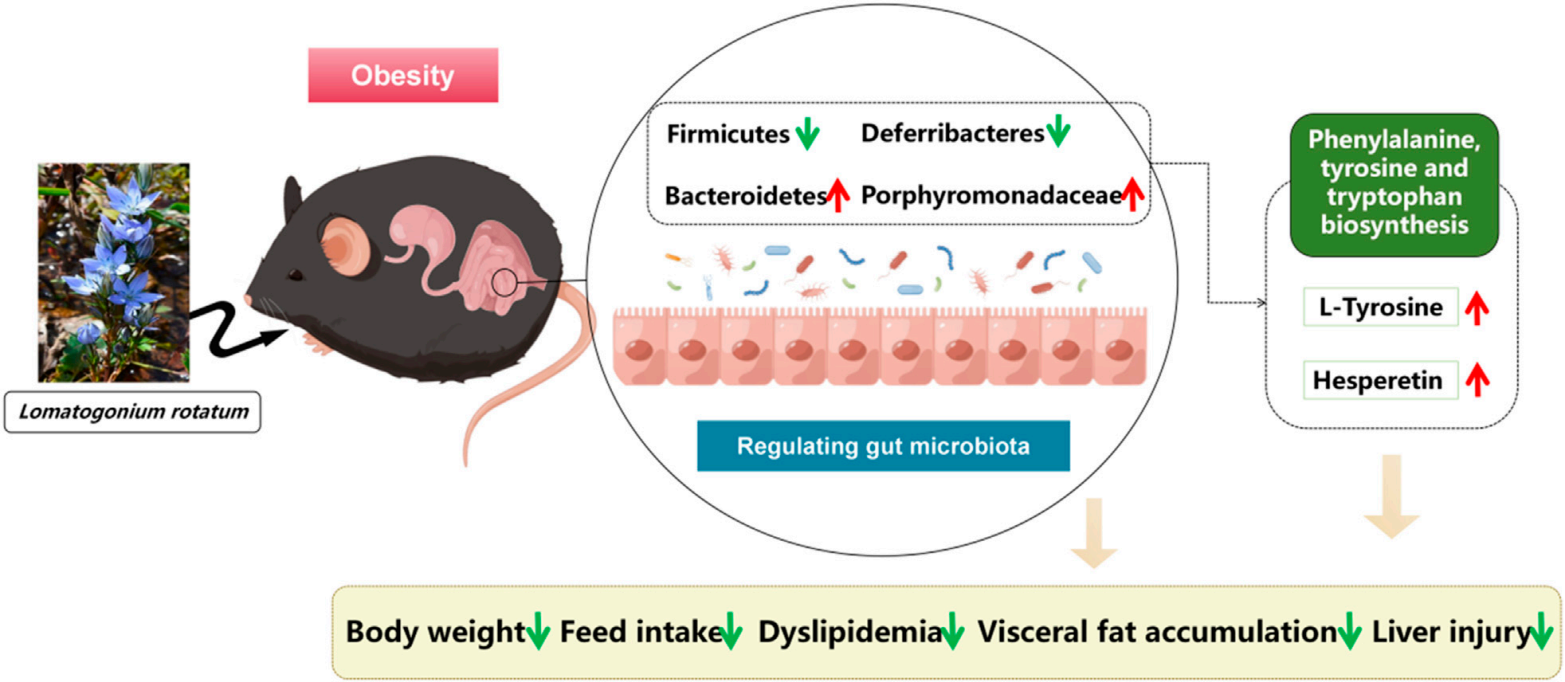
Mechanism diagram of LR for anti-obesity, visceral fat and lipid reduction.

Overall, LR exhibited significant potential in improving obesity in mice induced by a high-fat diet through several molecular mechanisms. At the gut microbiota level, LR caused a reduction in *Firmicutes* and *Deferribacteres*, while promoting an increase in *Bacteroidetes* and *Porphyromonadaceae*. This alteration in the microbial composition has profound implications. *Firmicutes* are often associated with enhanced energy extraction from the diet, contributing to obesity (Fan et al., 2021). The decrease in their abundance by LR might disrupt this excessive energy acquisition. *Deferribacteres*, on the other hand, have been implicated in various metabolic dysregulations (Tavella et al., 2021). By reducing their presence, LR may alleviate associated complications. The increased abundance of *Bacteroidetes* and *Porphyromonadaceae* is beneficial, as they are involved in promoting healthy metabolic processes (Stojanov et al., 2020). Furthermore, the increase in metabolites such as L-Tyrosine and Hesperetin, along with the enhanced biosynthesis of phenylalanine, tyrosine, and tryptophan, plays a crucial role (Taheri et al., 2023; Olsson et al., 2021). L-Tyrosine is integral to multiple metabolic pathways and neurotransmitter synthesis, influencing energy expenditure and appetite control. Hesperetin, with its antioxidant and anti-inflammatory properties, can counteract the inflammatory processes often associated with obesity (Figure 9). In terms of visceral and serum lipid metabolism, we speculate that LR’s effects may be mediated through molecular signalling pathways. For instance, LR may influence the PPARγ (Peroxisome Proliferator-Activated Receptor gamma) signalling pathway. Activation of PPARγ can lead to increased fatty acid oxidation and decreased lipogenesis in visceral adipocytes, thereby reducing fat accumulation (Wang et al., 2023). Additionally, LR might modulate the AMPK (AMP-activated Protein Kinase) pathway, which is a key regulator of energy metabolism. Activation of AMPK can enhance glucose uptake and fatty acid oxidation, contributing to the improvement of serum lipid profiles (Huang et al., 2021). Collectively, these molecular-level alterations orchestrated by LR ultimately lead to the amelioration of visceral fat accumulation and improvement in serum lipid profiles, presenting a promising therapeutic approach for obesity and related metabolic disorders. However, further in-depth studies are necessary to fully understand and validate these mechanisms.

## 5 Conclusion

In summary, LR treatment positively modulates weight gain, visceral fat accumulation in HFD-induced obesity, reduces food intake, and modulates dyslipidemia and liver injury. In addition, LR modulated disorders of gut microorganisms such as *Bacteroidetes*, *Firmicutes*, *Deferribacteres* and *Porphyromonadaceae*. These characterizations may be related to changes in relevant metabolites influenced by gut flora, such as the metabolism of L-Tyrosine and, hesperetin, which in turn act as anti-obesity agents. Our findings provide new insights into the mechanisms underlying the effects of LR on obesity and offer new avenues for its therapeutic intervention.

## Data Availability

The data presented in the study are deposited in the NCBI BioProject repository, accession number PRJNA1099761. Available at: https://www.ncbi.nlm.nih.gov/search/all/?term=PRJNA1099761.
